# A novel metastatic tumor segmentation method with a new evaluation metric in clinic study

**DOI:** 10.3389/fmed.2024.1375851

**Published:** 2024-10-02

**Authors:** Bin Li, Qiushi Sun, Xianjin Fang, Yang Yang, Xiang Li

**Affiliations:** ^1^Department of Neurology, The First Hospital of Anhui University of Science and Technology, Huainan, China; ^2^Department of Anesthesiology, Fudan University Affiliated Huashan Hospital, Shanghai, China; ^3^Department of Anesthesiology, Fudan University Affiliated Huashan Hospital, Huainan, China; ^4^School of Safety Science and Engineering, Anhui University of Science and Technology, Huainan, China

**Keywords:** brain metastases, precise treatment, deep learning, medical image segmentation, multi-objective segmentation integrity metric

## Abstract

**Background:**

Brain metastases are the most common brain malignancies. Automatic detection and segmentation of brain metastases provide significant assistance for radiologists in discovering the location of the lesion and making accurate clinical decisions on brain tumor type for precise treatment.

**Objectives:**

However, due to the small size of the brain metastases, existing brain metastases segmentation produces unsatisfactory results and has not been evaluated on clinic datasets.

**Methodology:**

In this work, we propose a new metastasis segmentation method DRAU-Net, which integrates a new attention mechanism multi-branch weighted attention module and DResConv module, making the extraction of tumor boundaries more complete. To enhance the evaluation of both the segmentation quality and the number of targets, we propose a novel medical image segmentation evaluation metric: multi-objective segmentation integrity metric, which effectively improves the evaluation results on multiple brain metastases with small size.

**Results:**

Experimental results evaluated on the BraTS2023 dataset and collected clinical data show that the proposed method has achieved excellent performance with an average dice coefficient of 0.6858 and multi-objective segmentation integrity metric of 0.5582.

**Conclusion:**

Compared with other methods, our proposed method achieved the best performance in the task of segmenting metastatic tumors.

## 1 Introduction

Brain metastases (BM) represent the predominant intracranial malignancies, emanating from primary sources like breast cancer, melanoma, and other cancers (1). As a distinct pathological entity, the therapeutic approach to managing BM encompasses a multitude of options, including whole-brain radiation therapy, stereotactic radiosurgery, surgical resection, targeted therapy, and immunotherapy (2). Precise identification of BM assumes paramount significance for clinicians, facilitating the initial screening for intracranial lesions, formulating timely and tailored treatment strategies, and prognosticating follow-up responses to avert unfavorable clinical outcomes.

Owing to the diverse and complex nature of metastatic tumors, magnetic resonance imaging (MRI) technology emerges as a pivotal tool for elucidating the comprehensive landscape of these malignancies. Serving as a non-invasive imaging technique, MRI not only furnishes essential intracranial functional information but also enables clinicians and researchers to attain a more holistic understanding of tumor tissue characteristics and lesion nature, leveraging its high spatial resolution and multimodal advantages (3). The segmentation of metastatic tumors yields extensive three-dimensional data, enriching pathological research. Through this segmentation process, insights into the tumor’s shape, size, and distribution are garnered, providing crucial information for the formulation of personalized treatment plans. Evaluation of the impact of treatment on the tumor, achieved by comparing segmentation results at different time points, facilitates timely adjustments to treatment plans, enhancing clinical efficacy. Nevertheless, manual delineation of segmentation results by experts proves inefficient, and the inherent variability in outcomes due to differing subjective opinions among doctors necessitates a more standardized approach (4). Exploring automated segmentation methods not only streamlines the workload for radiologists but also mitigates result discrepancies arising from subjective interpretations (5, 6).

Deep learning (DL), leveraging its data-driven and end-to-end capabilities, has found extensive applications in medical image analysis (7–9). Capitalizing on highly adaptive feature learning and multimodal fusion, deep learning-based frameworks exhibit a commendable ability to accurately delineate tumor boundaries (10–12). Numerous models have been previously proposed for quantitative analysis of BM but there still exist several challenges that hamper the clinical applicability of automatic detection (13). The first common challenge is boosting the detection of the small volume of BM and collaboratively decreasing false-positive (FP) rate (14). For experienced radiologists, detecting minuscule lesions presents a significant challenge, and any lesions that go unnoticed can substantially hinder the accuracy of patient diagnoses. The trade-off between the sensitivity and FP rate often puzzles the researchers in the Deep learning model design and selection (15). Models with high sensitivity would be inclined to identify and preselect the subtle lesions, whereas high FP would impede the accuracy of diagnosis. Yoo (16) proposed a DL model with a 2.5D overlapping patch technique to isolate a BM of less than 0.04cm^2^ in CE-MRI. Their model could detect relatively small tumors compared to previous studies, but the overall dice accuracy of the model is not satisfactory. Dikici (17) used a dual-stage framework to enhance the precision of isolating small lesions with an average volume of only 159.6 mm^3^. The framework, consisting of the candidate-selection stage and detection stage with a custom-built 3D CNN, achieves a high sensitivity on their BM database. However, due to model parameter limitations, it cannot recognize lesions exceeding 15 mm. Furthermore, the accuracy of BM detection and segmentation is limited by the characteristics and the quality of MRI images (18). In addition, different MRI imaging equipment and sequence parameters pose considerable challenges to the generalization ability of segmentation models. Zhou (19) trained a DL single-shot detector based on T1-weighted gradient-echo MRI, and the sensitivity of the testing group was 81% but only validated in single-center data. Grøvik (20) used four different MRI sequences for segmentation using the DeepLab V3 network, achieving a dice accuracy of 0.79. Although validated in two central data sets, the overall sample size is only 100 cases. In summary, the accuracy of existing segmentation methods for metastatic tumors is low, and their performance in clinical applications is inadequate. Therefore, developing a new segmentation technique for brain tumors that can precisely segment small metastatic tumors and deliver improved results, even with limited resolution, remains a challenging problem (21, 22).

In the context of medical image segmentation, metrics such as dice similarity coefficient metric and intersection over union not only gauge the accuracy of the segmentation model but are also frequently employed as loss functions for the model. However, current segmentation evaluation metrics often assess segmentation at a global level, which presents certain limitations. For instance, the dice similarity coefficient metric is sensitive to larger segmentation regions when assessing the presence of multiple segmentation regions within an image. Consequently, this approach may not provide an objective evaluation of smaller targets.

To enhance segmentation model generalization, we introduce an encoder-decoder framework incorporating deep convolution and attention mechanisms. Using publicly available brain imaging data for model training, and evaluate and compare performance metrics against various existing segmentation models in this study. The results show that our proposed model has excellent segmentation ability.

The main contributions of this article are as follows:

A new BM segmentation method for effectively extracting tumor boundaries and features: We propose DRAU-Net, a novel medical image segmentation method incorporating a multi-branch weighted attention module and multiple dilated residual convolution modules. This method achieved accurate segmentation results, demonstrating robust performance across a range of clinical medical settings, including those involving low-quality images and datasets with limited layers.

It is the first time an indicator that focuses on the global situation is proposed: In order to solve the problem of quantitatively calculate the lots of BMs with small sizes. This article first proposes a new medical image segmentation evaluation metric: multi-objective segmentation integrity metric (MSIM), which evaluates the integrity of multiple segmentation targets, a metric overlooked by most existing indicators.

We are validating the effectiveness of DRAU-Net on multiple datasets: To assess the generalization capability of our proposed segmentation method across diverse datasets, we have obtained favourable results from both publicly available and collected clinical metastasis datasets.

The structure of this article is organized as follows: The introduction, presented in the first section, highlights the clinical significance of metastasis segmentation and delineates the challenges currently faced in this field. The Materials and Methods section of the second section, details the brain tumor segmentation dataset utilized, outlines the data preprocessing procedures, introduces the novel DRAU-Net segmentation approach, and describes the experimental details. In section three, Experiment and Results, we present and analyse the segmentation evaluation indicators, comparative experiments, and ablation experiments of this article. The fourth section of the discussion, deliberates on the methodologies proposed within this work and offers insights into potential future directions. Finally, we summarized this article in section five.

## 2 Materials and methods

### 2.1 Data introduction

To assess the model generalization ability across diverse datasets, we employed the BraTS2023 Brain Metastases dataset, which encompasses data on brain metastases acquired from various institutions under different standard clinical conditions (23, 24). As the BraTS2023 test dataset details were not disclosed, we introduced randomness by shuffling the remaining data. Subsequently, 210 samples were designated for the training set, while 28 samples were set aside for the testing set. The BraTS2023 comprises multi-parameter MRI scans, including pre-contrast T1-weighted (t1w), post-contrast T1-weighted (t1c), T2-weighted (t2w), and T2-weighted Fluid Attenuated Inversion Recovery (t2f) images. All MRI images underwent standardization, co-registration to the common analytical template (SRI24), and skip striping. For segmentation purposes, the BraTS2023 Brain Metastases dataset utilizes three labels: No-enhancing tumor Core (NETC; Label 1), Surrounding non-enhancing FLAIR hyperintensity (SNFH; Label 2), and Enhancing Tumor (ET; Label 3).

**Figure 1:**
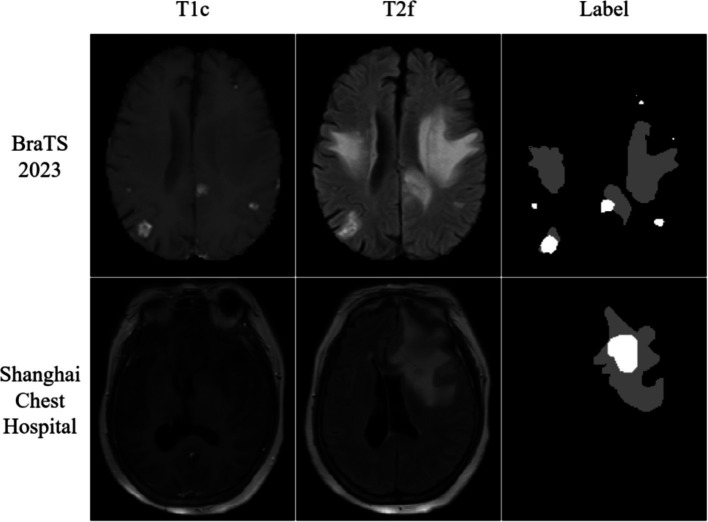
Visualization of the public dataset with the actual dataset we obtained.

Furthermore, this study conducted experiments using the metastasis dataset generously provided by Shanghai Chest Hospital. This dataset encompasses metastatic data from a cohort of 103 patients, acquired through the utilization of the 1.5 T MRI system (SignA Elite HD; GE Healthcare, Milwaukee, WI, USA). The dataset includes T2 Fluid Attenuated Inversion Recovery (T2 Flair) and post-contrast T1-weighted (T1ce) images. Within the context of brain metastases, segmentation is facilitated by two distinct labels: the whole tumor division label (WT) and the tumor core division label (TC). The visualization results of the dataset are shown in Figure 1.

### 2.2 Data preprocessing

In the case of the BraTS2023 Brain Metastases dataset, we opted for post-contrast T1-weighted (t1c) and T2-weighted Fluid Attenuated Inversion Recovery (t2f) as the inputs for our network. To maintain label consistency, we employed the whole tumor division label (Label 2) and the tumor core division label (Label 1 + Label 3).

In accordance with the data supplied by Shanghai Chest Hospital, Flair and T1ce image data underwent regularization using the Z-Score method prior to their integration into the network. Considering that the background in medical images does not provide useful information for segmentation, crop the image to the center region of 160 × 160 and normalize it.

### 2.3 Deep learning network method

**Figure 2:**
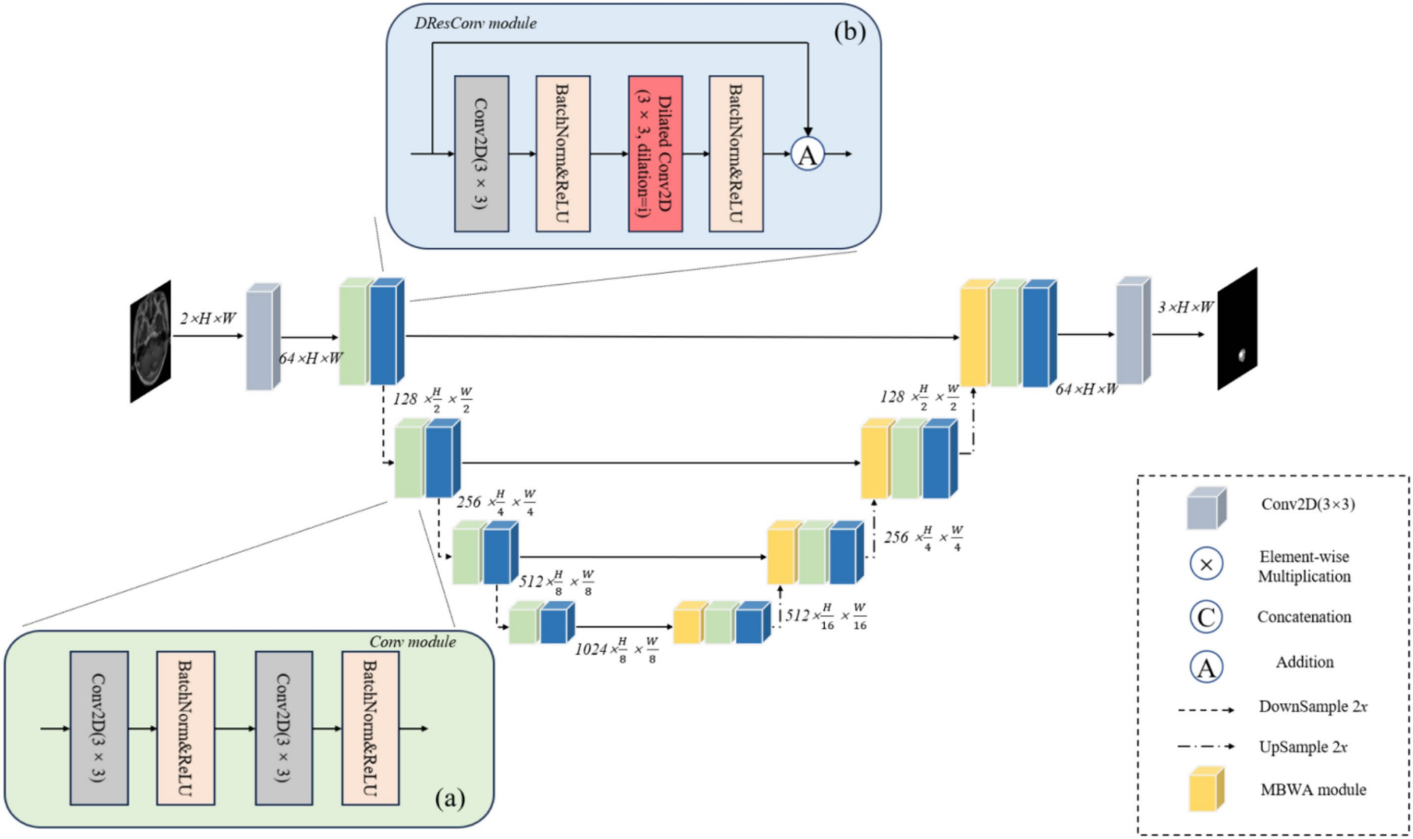
The illustration of DRAU-Net proposed for automatic brain metastasis of tumors. **(A)** Shows the flow chart of Conv module. **(B)** Shows the flow chart of DResConv module.

To attain precise segmentation of medical images with high accuracy, we consider that segmentation models should amalgamate focalization through convolution and attention mechanisms. Hence, we introduce a coding-decoder structured framework named DRAU-Net. Drawing inspiration from ResU-Net (25), as shown in Figure 2. Our architecture incorporates four convolution blocks on the encoder path. Post each convolution block, a DResConv module is employed to augment the network expressive capacity, yielding features with varying resolutions through downsample. In the decoder module, we introduce a novel attentional mechanism termed the MBWA module, designed to capture key features across the entire tensor. Subsequently, upsample is achieved through the convolution block and DResConv block, culminating in the final segmentation result. In the subsequent sections, a detailed account of each component’s specific implementation will be provided.

#### 2.3.1 Conv module

As shown in Figure 2A. The convolution block comprises two convolution layers, each featuring a 3 × 3 convolution kernel size and a stride of 1. In the implementation, following each convolutional layer, batch normalization and rectified linear units (ReLU) are applied. Downsample is employed to acquire raw images of diverse sizes, effectively diminishing the computational load of the model, mitigating overfitting, and enhancing the receptive field. This approach not only reduces computational complexity but also promotes a broader sensing field, enabling the subsequent module to effectively capture global information during the learning process.

#### 2.3.2 DResConv module

ResNet successfully addresses the challenge of gradient vanishing during deep network training by introducing residual blocks (26). However, comprehending global information without introducing extra parameters remains a critical issue. As illustrated in Figure 2B, we incorporate dilated convolutions with varying dilation rates into the residual block to expand the receptive field without introducing additional parameters, thereby enhancing the model’s ability to understand global features. Furthermore, convolution layers with distinct dilation rates effectively preserve local details within the image. This facilitates the network in learning a sparser representation of features, thereby capturing the structural information of the image more effectively. The specific implementation process is detailed as follows Equations (1–2):


(1) $$F=\mathrm{Conv}2D_{\mathrm{kenel}=3,\mathrm{didated}=1}\left(\mathrm{input}\right)$$

(2) $$\mathrm{output}=\mathrm{Conv}2D_{\mathrm{kernel}=3,\mathrm{dilated}=i}\left(F\right)+\mathrm{input}$$

Where input and output are inputs and output results, $\mathrm{Conv}2D_{\mathrm{kernel}=3,\mathrm{dilated}=i}$ represents a 2D convolution with a convolution kernel size of 3 and a void rate of i, where i is the number of layers.

#### 2.3.3 MBWA module

**Figure 3:**
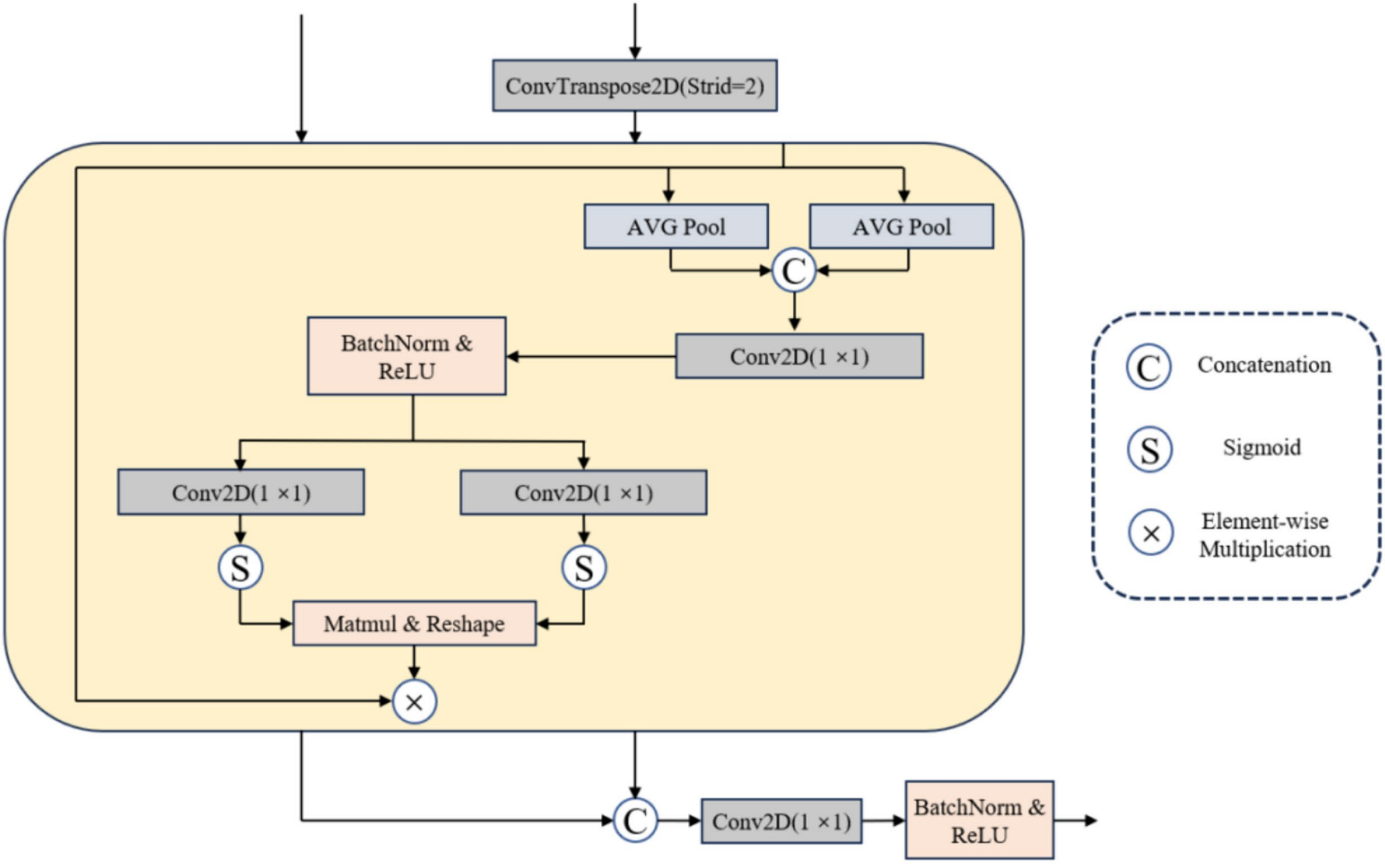
Flow chart of MBWA module.

Confronted with the challenge of multi-modal metastatic tumor segmentation, while the skip connection in the U-Net network facilitates information flow and transmission (27), it may inadvertently introduce redundant information. Achieving model focus on the target area becomes a significant challenge. In response, this article proposes an effective multi-branch weighted attention (MBWA). As illustrated in Figure 3, the MBWA module incorporates skip connections and utilizes the feature map from the decoder section of the preceding layer as input. Initially, it adjusts the resolution of the decoder features from the previous layer through a 1 × 1 transposed convolution. Subsequently, these adjusted features undergo weighted attention coordination within the MBWA module before being concatenated. Finally, a 1 × 1 convolution is applied to adjust the channel dimensions.

The MBWA module is implemented as follows: First, we carry out the maximum average pooling of the output tensor in horizontal dimension and vertical dimension respectively Equations (3–4):


(3) $$x_{1}=AVG_{\mathrm{horizontal}}\left(I\right)$$

(4) $$x_{2}=AVG_{\mathrm{vertical}}\left(I\right)$$

I denote the output tensor, while $AVG_{\mathrm{horizontal}}$ and $AVG_{\mathrm{vertical}}$ symbolize the average pooling of the feature tensors along the horizontal and vertical dimensions, respectively. Employing global pooling along the horizontal dimension enables feature interaction in the spatial domain. This process preserves the positional information $x_{1}$ along the horizontal dimension, yielding the spatial attention weight. Meanwhile, $x_{2}$ retains long-range dependencies along the vertical dimension and captures positional information in that axis. By concatenating the obtained horizontal and vertical spatial coding information, we subsequently input this combined information into a 1 × 1 convolution layer. This step facilitates the extraction of meaningful spatial features by integrating both horizontal and vertical position information Equation (5).


(5) $$\tilde{x}=\mathrm{ReLU}(BN\left(\mathrm{Conv}2D_{\mathrm{kernel}=1}\left(x_{1},x_{2}\right)\right)$$

ReLU represents the rectified linear unit activation function, BN denotes the batch normalization operation, and $\mathrm{Conv}2D_{\mathrm{kernel}=1}$ signifies a 2D convolution with a convolution kernel size of 1. Following the acquisition of horizontal and vertical spatial coding information $\tilde{x}$ , a split separation is executed, and the weight map is generated by reinstating the channel count through two 1 × 1 convolutions. Ultimately, the weights are aggregated and applied as weights to the original input Equations (6–9):


(6) $$x_{h},x_{v}=\mathrm{Split}\left(\tilde{x}\right)$$

(7) $$w_{h}=\sigma (BN\left(\mathrm{Conv}2D_{\mathrm{kernel}=1}\left(x_{h}\right)\right)$$

(8) $$w_{v}=\sigma (BN\left(\mathrm{Conv}2D_{\mathrm{kernel}=1}\left(x_{v}\right)\right)$$

(9) $$O=I\times \left(w_{h}+w_{v}\right))$$

Split denotes separation along spatial dimensions, σ represents the sigmoid activation function, BN stands for normalization operation, and $\mathrm{Conv}2D_{\mathrm{kernel}=1}$ represents a 2D convolution with a convolution kernel size of 1. The MBWA module incorporates positional and spatial information from the input tensor into the output result. The feature coordination across different dimensions within the MBWA module not only tailors the output result to dynamically adjust channel weights but also introduces long-range dependencies in the spatial dimension, thereby enhancing the network’s attention to critical features. This diminishes redundant information and augments the network’s representational capability.

### 2.4 Loss function

Due to the large number of brain metastases and small lesions, we use a combined loss function to constrain the optimization direction of the model and further improve the segmentation results. The loss is given by the following formula Equations (10–12):


(10) $$\mathrm{Loss}=\mathrm{\alpha Los}s_{\mathrm{Dice}}+\mathrm{\beta Los}s_{BCE}$$

(11) $$Loss_{BCE}=-\left[\mathrm{Tlog}\left(P\right)+\left(1-T\right)\log\left(1-P\right)\right]$$

(12) $$Loss_{\mathrm{Dice}}=1-\frac{2|P\cap T|}{\left|p|+|T\right|}$$

Where T represents the ground truth, P represents the segmentation result, α,β represents the weight of $Loss_{BCE}$ and $Loss_{\mathrm{Dice}}$ . In this study, α,β are set to 0.7 and 0.3, respectively.

### 2.5 Implementation details

All the experiments from different models were implemented on the server with the following framework: one 12-core Intel 12,700 K CPU, one NVIDIA 3080Ti GPU (12GB), and 32GB RAM. We implement all models on PyTorch. All experimental and comparison models do not use any pre-trained models already trained. The models are trained using the Adam optimizer with an initial learning rate of 3 × 10^−4^, the batch size of 8, and the training epoch is set to 150.

## 3 Experiments and result

### 3.1 Evaluation metrics

The model is evaluated using several commonly employed medical image segmentation metrics. The Dice coefficient metric measures the degree of similarity between two samples. Sensitivity measures the proportion of the sample that is correctly segmented. Positive predictive value (PPV) is the proportion of correctly predicted samples among all predicted samples. The lesion number indicator quantifies the proportion of correctly segmented lesions in the entire dataset. The Jaccard index is used to evaluate the intersection-over-union coefficient, as given below Equations (13–16):


(13) $$\mathrm{Dice}=\frac{2|p\cap t|}{\left|p|+|t\right|}$$

(14) $$\mathrm{Sensitivity}=\frac{TP}{TP+FN}$$

(15) $$PPV=\frac{TP}{TP+FP}$$

(16) $$\mathrm{Jaccard}=\frac{\left|p\cap t\right|}{\left|p\cup t\right|}$$

Here, p represents the ground truth, t represents the segmentation result. TP , FP and FN indicate true-positive, false-positive, and false-negative predictions.

Commonly used medical image segmentation metrics are typically evaluated based solely on the degree of overall image segmentation. However, this evaluation criterion has several limitations. For instance, in the case of the dice, the degree of the larger segmentation region has a greater impact on the overall segmentation metric when multiple segmentation regions in the image are evaluated. As a result, this approach is unable to provide an objective assessment of smaller targets. In the context of metastasis segmentation, the small size of the metastases and the completeness of their segmentation are critical factors that need to be taken into account. To address this issue, we propose a novel segmentation evaluation metric called the multi-objective segmentation integrity metric.

#### ALGORITHM 1 Multi-objective segmentation integrity metric

**Table:** 

**Input:** $\mathrm{Labe}l_{gt}$ , $\mathrm{Labe}l_{pre}$ **Output:** $MSIM_{\mathrm{source}}$ **Define:** δ : threshold of area filtering; θ : threshold of dice source
1: Load $\mathrm{Labe}l_{gt}$ and $\mathrm{Labe}l_{pre}$ 2: Get connected domains: $g_{i}\leftarrow \mathrm{Labe}l_{gt}$ 3: Get connected domains: $p_{i}\leftarrow \mathrm{Labe}l_{pre}$ 4: **for** k=0→i−1 **do** 5: Get connected domain size $s_{k}\leftarrow g_{k}$ 6: **if** $s_{k}<\delta$ or do not have tumor core label **then** 7: Delete $g_{k}$ 8: **end if** 9: Morphological dilation of $g_{k}$ 10: Morphological erosion of $g_{k}$ 11: **end for** 12: Sort $g_{i}$ , $p_{i}$ according to the size of the connected domain
13: Get number of connected domains: $u\leftarrow g_{i}$ 14: Get number of connected domains: $v\leftarrow p_{i}$ 15: **for** j=0→u−1 **do** 16: σ← Calculate the dice metric of $g_{i}$ and $p_{i}$ 17: **if** σ>θ **then** 18: num + 1 19: **end if** 20: **end for** 21: **return** num / u

**Figure 4:**
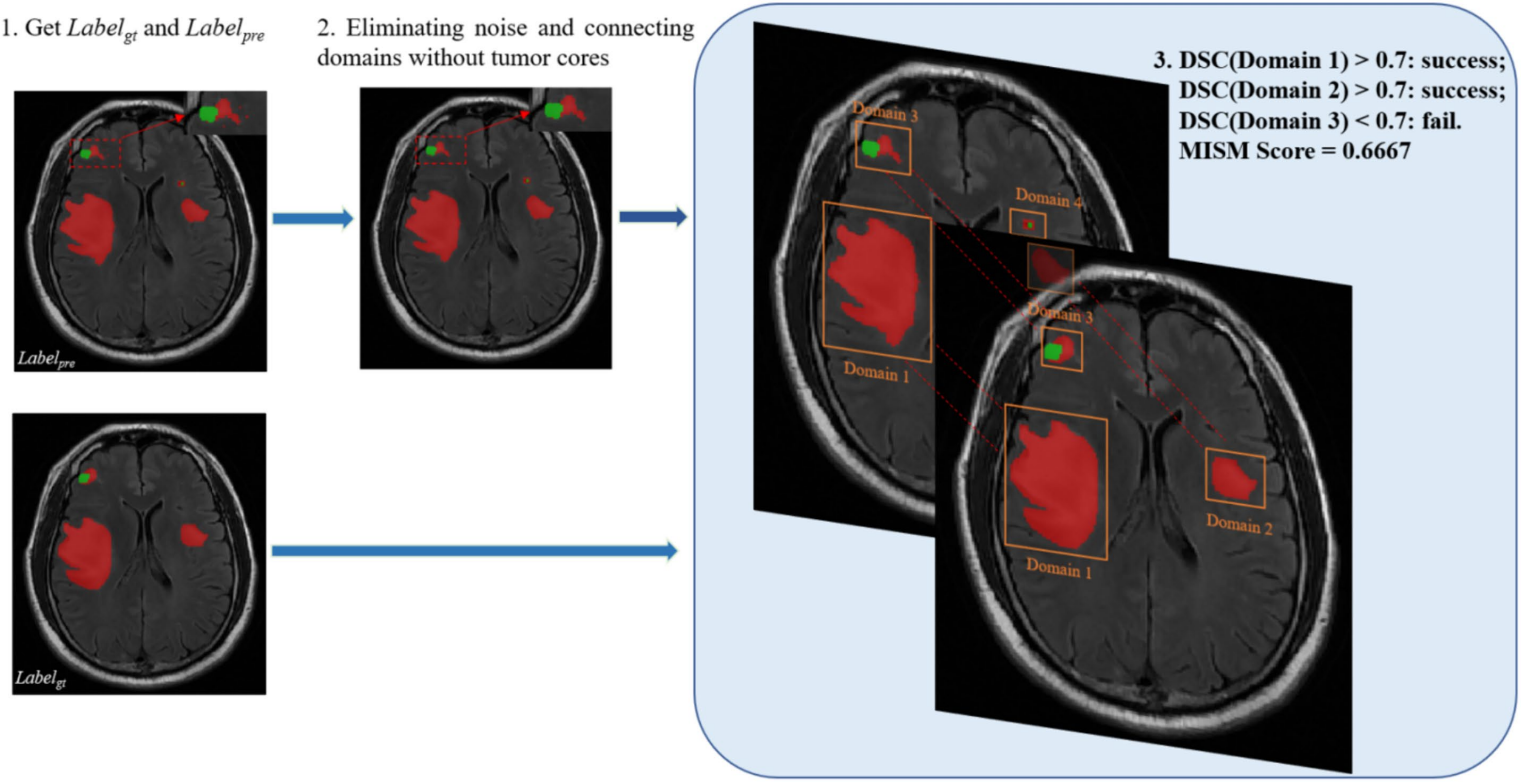
Flowchart of MSIM.

Algorithm 1 outlines the general workflow of our proposed MSIM. As shown in Figure 4, this evaluation metric involves obtaining all the segmented regions and comparing them with the true segmented regions in pairs, which enables the detection of the true segmentation of each region. The dice coefficient metric is used in the MSIM evaluation metric to determine whether each region has been successfully segmented, we use 0.7 as the success criterion. The final segmentation metric is calculated as the ratio of the number of successfully segmented regions to the total number of regions in the actual image segmentation. The specific calculation process is as follows Equations (17–18):


(17) $$S=\frac{2|V_{P}\cap V_{T}|}{\left|V_{P}|+|V_{T}\right|}>0.7$$

(18) $$\mathrm{MISM}=\frac{\sum_{i}S_{i}}{\sum_{j}D_{j}}$$

Where $V_{P}$ is the number of pixels in the predicted target domains, and $V_{T}$ is the number of pixels in the target domains in the ground truth. $S_{i}$ is the number of successfully segmented targets domains, and $D_{j}$ is the number of targets domains in the ground truth.

### 3.2 Comparison with other existing segmentation methods

To evaluate the effectiveness of our DRAU-Net, we compare DRAU-Net with other representative segmentation methods, including U-Net, DenseU-Net (28), AttU-Net (29), U-Net++ (30), U-Net3Plus (31), ResU-Net, TransU-Net (32), LMBiS-Net (33) and HSA-Net (34). All models compared in this study were evaluated without any pre-training strategy, model ensembling, or data augmentation techniques. The results in Table 1 show that the pure U-Net model based on CNN achieved a WT dice of 75.39. The DenseU-Net method, which uses dense layers, improves the U-Net and achieves a WT dice of 73.68. The U-Net++, which improves skip connections, outperformed other models in terms of PPV. Our proposed DRAU-Net achieves a 3.36 increase in dice compared to the most recent U-Net3Plus. In addition, DRAU-Net outperforms HAS-Net in the Sensitivity metric with a TC score of 77.02 and an ET score of 73.90. For the Jaccard metric, our proposed method leads other models with an average score of 65.18. Regarding the MSIM, the proposed evaluation metric for complete segmentation of BM, DRAU-Net performs the best among all models with a score of 48.80.

**Table 1:** The comparison results between the proposed method and other comparative experiments in BraTS2023 dataset.

Methods	Dice (%)↑		PPV (%)↑		Sensitivity (%)↑		Jaccard (%)↑		MSIM (%)↑
	WT	TC	ET	WT	TC	ET	WT	TC	
U-Net	75.39	66.40	**85.87**	75.21	70.01	67.04	64.56	55.55	47.61
DenseU-Net	73.68	70.73	81.48	75.10	69.72	68.92	63.45	60.00	40.47
AttU-Net	70.61	65.20	85.32	75.01	64.73	65.12	59.82	54.23	44.04
U-Net++	74.93	68.07	87.93	**78.07**	69.45	68.21	64.55	57.88	47.61
U-Net3Plus	75.79	68.96	84.14	74.82	70.84	68.87	65.69	58.41	45.23
ResU-Net	73.77	66.75	80.46	69.56	69.82	69.48	63.12	56.31	47.61
Res2U-Net	70.90	64.84	78.80	69.33	67.49	66.63	60.01	54.36	36.90
TransU-Net	70.18	64.35	80.69	71.04	64.97	64.46	59.38	53.44	36.90
LMBIS-Net	73.08	66.42	84.04	72.46	67.25	66.78	62.43	55.78	40.47
HSA-Net	78.38	69.46	84.70	74.67	75.13	70.10	68.33	59.12	47.61
**DRAU-Net**	**79.15**	**71.47**	84.13	74.42	**77.02**	**73.90**	**68.93**	**61.43**	**48.80**

Bold represents the optimal value.

**Figure 5:**
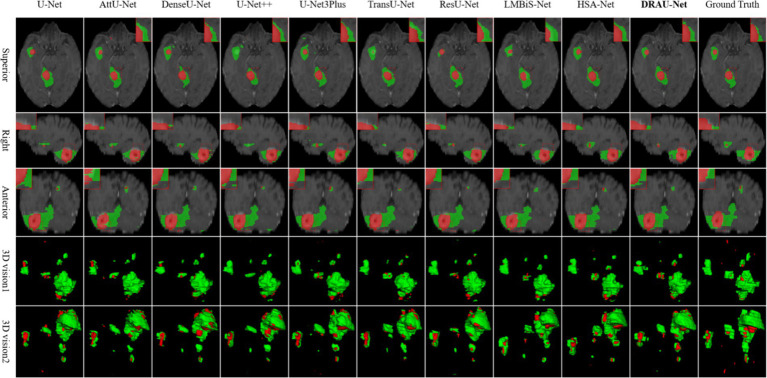
Visualize and compare the results with other models on the BraTS2023 dataset.

In Figure 5, the segmentation results of the compared models are presented from multiple dimensions, and the difficult-to-segment regions are highlighted using red dotted lines. It is evident that accurately segmenting the BM, especially the tumor core, remains a significant challenge for existing methods. Both AttU-Net and U-Net3Plus struggle to delineate the tumors boundaries.

accurately. In contrast, our DRAU-Net demonstrates improved segmentation accuracy. Furthermore, in Figure 5, we show that other methods may miss small tumors when segmenting multiple BM, whereas DRAU-Net accurately segments even small BM.

To further validate the effectiveness of our segmentation framework, this study verified the accuracy of WT and ET segmentation on the data of metastatic tumors provided by Shanghai Chest Hospital dataset. As shown in Table 2, DRAU-Net outshines others with its superior dice scores for whole tumor, achieving 69.52 and 68.95%, respectively. These scores not only surpass those of the well-established U-Net and its derivatives such as DenseU-Net, AttU-Net, and U-Net++, but also significantly outperform other latest models like LMBIS-Net and HSA-Net. Of particular note is that DRAU-Net achieved an average sensitivity and average PPV of 65.32 and 71.38, respectively. This indicates that DRAU-Net not only has a powerful ability to detect key tumor regions, but also can accurately segment key tumor regions, which is crucial for effective medical diagnosis and treatment planning. In addition, DRAU-Net achieved the best performance under the MSIM metric, indicating its robustness in overall segmentation of metastatic tumors. Compared to other segmentation methods, DRAU-Net also produces more stable segmentation results.

**Table 2:** The comparison results between the proposed method and other comparative experiments in Shanghai Chest Hospital dataset.

Methods	Dice (%)↑		PPV (%)↑		Sensitivity (%)↑		Jaccard (%)↑		MSIM (%)↑
	WT	TC	ET	WT	TC	ET	WT	TC	
U-Net	61.17	54.36	56.80	62.85	78.17	64.53	48.74	41.81	51.42
DenseU-Net	63.41	66.76	62.81	84.97	77.88	60.52	52.33	55.30	60.00
AttU-Net	61.21	**67.82**	57.46	83.58	77.00	62.19	48.35	**56.16**	54.28
U-Net++	65.90	62.95	64.06	**88.37**	74.41	54.53	53.19	50.99	51.42
U-Net3Plus	66.85	63.45	66.52	78.45	75.37	57.40	53.87	51.45	54.28
ResU-Net	59.52	67.03	54.38	88.71	74.66	59.75	47.01	56.06	54.28
TransU-Net	55.64	34.28	53.54	46.21	69.35	45.46	42.30	22.80	51.42
LMBIS-Net	64.65	56.63	61.54	66.16	**79.53**	**65.89**	51.69	43.66	51.42
HSA-Net	60.61	47.02	57.68	57.12	76.25	65.09	47.62	34.98	48.57
**DRAU-Net**	**69.52**	54.19	**68.95**	61.70	77.71	65.65	**56.48**	40.73	**62.85**

Bold represents the optimal value.

**Figure 6:**
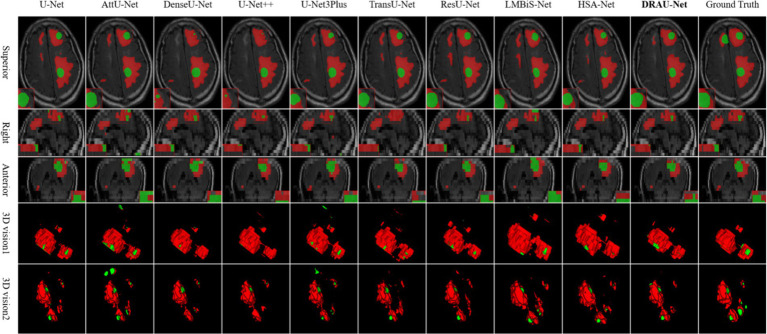
Visualize and compare the results with other models on the Shanghai Chest Hospital dataset.

In Figure 6, the segmentation results are visualized. Notably, DRAU-Net has the clearest boundary segmentation for WT and TC among all networks, as can be clearly seen from the enlarged red dotted line. Furthermore, this segmentation approach has the least amount of noise at the segmentation edge. Figure 6 presents the segmentation results from multiple dimensions. From the 2D slices, DRAU-Net achieves more accurate segmentation closer to the ground truth. In the two 3D views, DRAU-Net produces smoother boundaries, fewer surrounding noises, and more detailed segmentation results.

### 3.3 Ablation study

This research proposed DRAU-Net framework elaborates two learnable modules, including DResConv, MBWA. To verify their contributions in performance of the segmentation model, we conducted a series of ablation experiments. The arrangement of each module in the ablation experiments is shown in Table 3.

**Table 3:** Design scheme of ablation study.

Methods	DResConv	MBWA
Ablation 1		
Ablation 2		√
Ablation 3	√	
Ablation 4	√	√

In the context of multi-modal transfer tumors segmentation, attention mechanisms are crucial, as there are typically numerous transfer tumors that are relatively small compared to other tumors. The MBWA module enhances the model feature extraction ability by effectively integrating shallow convolutional features with deep features from the encoder, while simultaneously strengthening the model’s regions of interest. The DResConv module enhances the ability to extract global information by expanding the receptive field while extracting features. The combination of both modules strengthens the model’s feature extraction ability for small targets. The results of the ablation experiment are presented in Table 4, indicating that the MBWA and DResConv modules independently improve the WT dice index by 1.26 and 0.32, respectively. Furthermore, the combination of both modules improves the WT and TC dice coefficients by 2.14 and 1.48, respectively.

**Table 4:** Quantitative comparison of ablation results using various modules on BraTS2023 dataset.

Methods	Dice (%)↑		PPV (%)↑		Sensitivity (%)↑		Jaccard (%)↑		MSIM (%)↑
	WT	TC	ET	WT	TC	ET	WT	TC	
Ablation 1	77.01	69.99	83.96	75.15	73.50	70.79	66.65	59.13	73.37
Ablation 2	78.27	70.00	83.67	73.48	75.79	72.05	67.93	59.79	75.16
Ablation 3	77.33	70.00	83.87	73.48	75.79	72.05	67.93	59.79	75.00
Ablation 4	79.15	71.47	84.13	74.42	77.02	73.90	68.93	64.43	48.80

## 4 Discussion

Accurate and effective segmentation of BM lesions is essential for clinical diagnosis and prognosis evaluation. This study proposes a method that aims to segment multiple lesions in clinical data without relying on any pre-trained models. In the dataset of Shanghai Chest Hospital, the collected MRI data is highly heterogeneous. Thus, all images were resampled to 16 layers during preprocessing. However, the small number of layers may result in the loss of features of brain metastases on MRI, and some smaller metastases may be missed. Furthermore, due to time limitations, only two radiologists performed the ground truth labelling.

The performance of the proposed model was compared with other existing U-Net-based models on the BraTS2023 and Shanghai Chest Hospital datasets. In addition, we also studied and compared other deep-learning methods related to BM. Our proposed method and data are both yield good performance among similar methods.

In the ablation experiments, compared to the proposed model, Ablation 3 without MBWA and Ablation 2 without DResConv both show a significant decrease in the dice and PPV index of the segmentation task, indicating that both modules play an important role in the accuracy of model segmentation. In the experiments, we explore the application of Transformer-based attention mechanisms; however, the results are not satisfactory. Transformers require dividing the sequence into multiple subspaces, which may lead to different heads capturing similar or redundant information. The presence of redundant information can hinder the model’s ability to learn crucial features, resulting in decreased performance when dealing with clinical thin-layer BMs. DRAU-Net achieves the most balanced results on three indicators: dice, PPV, and sensitivity, confirming that the combination of the proposed attention mechanism and convolution is more helpful in segmenting brain metastases.

The large number and small size of brain metastases may mislead the segmentation result evaluation. As each segmentation target is critical, we propose MSIM to evaluate the complete segmentation of tumors. Based on the experimental results, the previous typical deep learning models such as U-Net also achieved a good result in our dataset based on dice. However, the MSIM of U-Net3Plus is 45.23, which is far below the result of our DRAU-Net with an MSIM of 48.80. More importantly, the disparity between the best and worst segmentation results, as measured by the dice, is merely 8.26. However, the difference between MSIM is 11.9. In comparison to the dice, the MSIM metric makes up for the deficiency of evaluation of multiple small lesions and results in superior evaluation performance.

However, the model has limitations due to the differences in image quality from different clinical centers. Firstly, due to the difficulty of data acquisition and cleaning, the data collected from Shanghai Chest Hospital in this study only included two modalities: T2 Flair and T1ce. This limitation resulted in slightly lower segmentation accuracy compared to public datasets. Moreover, since the format of multi-center datasets is often non-uniform, achieving a uniform size through resampling often leads to a loss of detail in the original images, reducing segmentation accuracy. Additionally, due to the limited availability of doctors and the extremely time-consuming process of labeling metastatic tumors, the clinical data in this study included only whole tumor division labels and tumor core division labels. This restriction has led to limited utility of the segmentation results for auxiliary diagnosis. In future work, the plan is to collect and expand the dataset by inviting more experts to annotate the data to reduce annotation errors and implement domain adaptation and data augmentation strategies to enhance segmentation accuracy.

## 5 Conclusion

As a secondary malignant tumor, metastatic tumors present significant challenges in clinical identification due to their complex shape, size, and distribution. In this paper, a multimodal automatic segmentation method for brain metastases based on the U-Net structure is proposed, designed to assist doctors in quickly identifying and locating brain metastases. This approach aims to optimize diagnosis and treatment plans, thereby improving patient outcomes. DRAU-Net captures more remote dependency information through the DResConv module, enhancing the feature extraction capability for small targets. The MBWA module integrates positional and spatial information from the images into the segmentation results, reducing redundant information while increasing focus on critical features. DRAU-Net has been validated on several datasets, demonstrating superior segmentation results compared to mainstream segmentation methods. Additionally, this research introduces the multi-objective segmentation integrity metric, which emphasizes the segmentation integrity of small target regions within multi-target tasks, providing a more objective evaluation for complex segmentation challenges such as BM segmentation.

In the future, the plan is to further optimize the DRAU-Net algorithm by exploring more efficient convolutional operations and attention mechanisms to enhance the model’s robustness. Additionally, domain adaptation and diffusion models will be incorporated to extend the application of DRAU-Net to other types of tumors and complex lesion segmentation. Finally, multi-center clinical trials will be conducted to verify the performance of DRAU-Net across different clinical settings and devices, ensuring its reliability and applicability in practical applications.

## Data Availability

The original contributions presented in the study are included in the article/supplementary material, further inquiries can be directed to the corresponding authors.
